# Activation of PI3K in response to high glucose leads to regulation of SOCS-3 and STAT1/3 signals and induction of glomerular mesangial extracellular matrix formation

**DOI:** 10.18632/oncotarget.14808

**Published:** 2017-01-24

**Authors:** Meei-Ling Sheu, Chin-Chang Shen, Jia-Rong Jheng, Chih-Kang Chiang

**Affiliations:** ^1^ Department of Medical Research, Taichung Veterans General Hospital, Taichung, Taiwan; ^2^ Institute of Biomedical Sciences, National Chung Hsing University, Taichung, Taiwan; ^3^ Rong Hsing Research Center for Translational Medicine, National Chung Hsing University, Taichung, Taiwan; ^4^ Chemical Engineering Division, Institute of Nuclear Energy Research, Atomic Energy Council, Taoyuan, Taiwan; ^5^ Institute of Toxicology, National Taiwan University College of Medicine, Taipei, Taiwan; ^6^ Department of Internal Medicine, National Taiwan University College of Medicine, Taipei, Taiwan; ^7^ Department of Integrated Diagnostics & Therapeutics, National Taiwan University Hospital, Taipei, Taiwan

**Keywords:** extracellular matrix, high glucose, mesangial cell, phosphoinositide 3-kinase, signal transduction

## Abstract

Excessive deposition of extracellular matrix (ECM) in the glomerulus contributed by mesangial cells is the hallmark of diabetic nephropathy, eventually leading to glomerulosclerosis. In this study, we examined the regulatory signals involved in the high glucose (HG)-induced overproduction of ECM in rat mesangial cells (RMCs). We disclosed excessive fibronectin and collagen IV production, tyrosine phosphorylation of signal transducer and activator of transcription 1 and 3 (STAT1/3), and up-regulation of suppressor of cytokine signaling-3 (SOCS-3) expression in HG-treated RMCs. STAT1/STAT3 binding element was essential for SOCS-3 promoter activity stimulated by HG. HG was capable of promoting the specific DNA binding activities to an oligonucleotide probe containing the SOCS-3 sequence. The selective phosphoinositide 3-kinase (PI3K) inhibitor LY294002 and dominant negative p85 vector (DNΔp85) transfection effectively abolished these HG-induced responses. Moreover, HG markedly increased the cyclin kinase inhibitor p27^Kip1^ protein expression, which could be inhibited by LY294002 or transfection of DNΔp85. Taken together, these results suggest that HG-induced SOCS-3 upregulation depends upon the presence of STAT-binding element in the SOCS-3 promoter, which is specifically activated by STAT1/3. The PI3K/STAT1/3 signaling pathway mediated HG-triggered ECM accumulation and SOCS-3 upregulation in RMCs.

## INTRODUCTION

Excessive accumulation of extracellular matrix (ECM) in the glomerulus is the hallmark of diabetic glomerulopathy and a key mechanism in the progression of glomerulosclerosis [1, 2]. Multiple intracellular regulators are thought to be involved in pathophysiology of ECM. One of the mechanisms for hyperglycemia-induced mesangial cell cycle arrest [3] and ECM accumulation [4] may be through the activation of signal transducer and activator of transcription (STAT) or more distinctive molecules recruited. In diabetes, the molecular basis of ECM accumulation is still an enigma, although new gene expression, protein synthesis, and matrix deposition have been linked to pathologic changes in glomerular mesangial cells [2, 5, 6]. High glucose (HG) has been shown to trigger the reactive oxygen species-regulated NF-κB activation and cyclooxygenase-2 expression and mesangial cell proliferation through a phosphoinositide 3-kinase (PI3K)-dependent pathway in early stage of HG exposure to mouse mesangial cells [7]. Similarly, it has been found that late-onset, stable changes in mesangial cell hypertrophy induction requires the activation of ERK, JNK/SAPK, and PI3K pathways [8]. However, the affection in gene regulation, which occurs during biological responses for diabetic glomerular ECM deposition, is still unclear. Moreover, the role of PI3K in the diabetic glomerular ECM accumulation has not been completely established.

SOCS-3 (suppressor of cytokine signaling-3) is an intracellular protein, refers to cytokine-inducible SH2-containing protein-3 (CIS-3), and belongs to the SOCS family of proteins. SOCS-3 is selectively and rapidly induced by appropriate cytokines and modulates responses of immune cells to cytokines by interfering with the Janus kinase (Jak)/STAT pathway [9, 10]. SOCS-3 expression is transiently induced by a wide variety of inflammatory and anti-inflammatory cytokines, including interferon (IFN)-γ, IL-3, IL-6, and IL-10 [11–15]. The specific antisense oligonucleotides inhibited mesangial SOCS-3 expression have been demonstrated to lead to an increase in the immune complex-induced STAT activation, and implicating that SOCS may be an important modulator of cell activation during renal inflammation [16]. Overexpression of SOCS-1 in human mesangial cells has also been found to inhibit HG-induced JAK2/STAT activation and increase TGF-β1 and fibronectin synthesis [17]. Ortiz-Muñoz et al. have suggested that a JAK/STAT/SOCS axis contributes to hyperglycemia-induced cell responses in the kidney [18]. The pathological role of mesangial cells in diabetes-induced nephropathy is well known, but poorly understands the alteration in the transcriptional gene regulation in mesangial cells under diabetic condition. In this study, we tried to elucidate whether SOCS-3 expression in mesangial cells can be induced by hyperglycemia condition, and to investigate the relationship among PI3K, STAT1/3, and SOCS-3 signaling in mesangial cells under HG condition, and to clarify the role of these signals in HG-induced mesangial ECM formation.

## RESULTS

### HG-induced ECM production of rat glomerular mesangial cells requires PI3K

**Figure 1:**
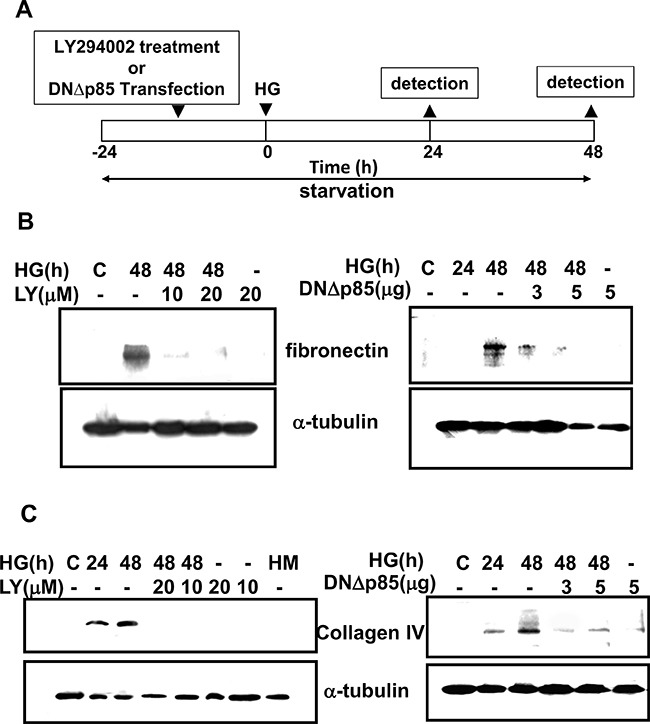
Induction of extracellular matrix proteins by high glucose in RMCs **A**. The figure described the time course of treatment for induction of extracellular matrix (ECM) formation. Briefly, RMCs were grown to around 80% confluence, washed once with serum-free DMEM, and growth-arrested in normal glucose serum-free DMEM to synchronize the cell growth. It followed by LY294003 pretreatment or dominant negative p85 vector (DNΔp85) for 16 hours before the treatment of high glucose (HG). The samples were examined at the indicated time points. **B**. and **C**. PI3K was required for high glucose (HG)-induced rat mesangial cell ECM accumulation. Mesangial cells were treated with serum-free DMEM containing normal glucose (5.5 mM) or HG (33 mM) for 24-48 h in the presence or absence of specific PI3K inhibitor (LY294002) or dominant negative p85 vector (DNΔp85) transfection. Fibronectin (B) and collagen IV (C) were analyzed by Western blotting. Results shown are representative of four independent experiments.

**Figure 2:**
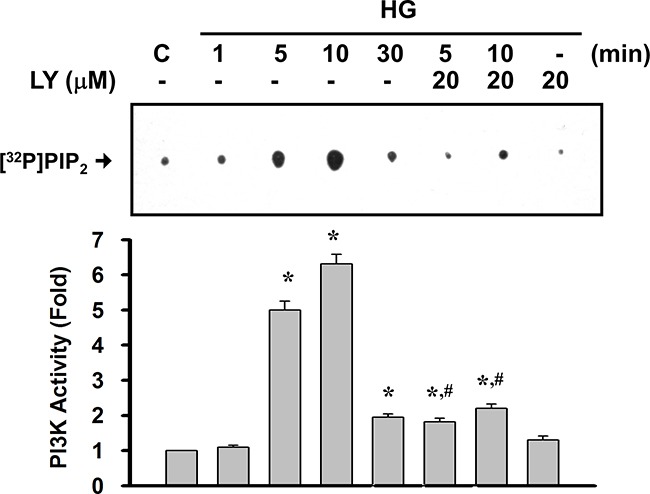
High glucose activated the PI3K activity in RMCs RMCs were treated with high glucose (HG) in the presence or absence of LY294002 (LY, 10 μM) for the indicated times. Cell lysates with equal amounts of protein were subjected to immunoprecipitations with anti-p85 PI3K subunit antibody. The immunocomplex was employed for PI3K activity assays as described under “Materials and Methods”. Quantification of the activity of PI3K was performed by densitometric analysis. Data are presented as means±SEM from three independent experiments performed in duplicate.

We first confirmed the role of PI3K signaling in the expression of matrix components, such as collagen IV and fibronectin, in primary rat mesangial cells (RMCs) under HG (33 mM) treatment. The PI3K signaling was disrupted by pharmacological selectively inhibitor (LY294002) or dominant negative p85 (DNΔp85) vector transfection. The treatment protocol for these experiments in RMCs was showed in Figure 1A. HG markedly triggered the protein expressions of fibronectin and collagen IV in mesangial cells (Figure 1B and 1C). Unlike HG, the addition of high mannitol (HM) to the media did not affect the expression of collagen IV in mesangial cells as compared with the control (Figure 1C). LY294002 and DNΔp85 transfection could effectively abolish HG-induced fibronectin and collagen IV protein expressions (Figure 1B and 1C). Furthermore, mesangial cells stimulated with HG triggered a rapid and transient activation of PI3K, reaching maximal level at 10 min and declining thereafter to background levels within 30 min (Figure 2). LY294002 significantly inhibited HG-increased PI3K activity. These results confirmed that PI3K activation and subsequent ECM accumulation forms the basis of a potential intracellular signaling response to HG in primary RMCs.

### HG-induced SOCS-3 expression and promoter binding activity requires PI3K

**Figure 3:**
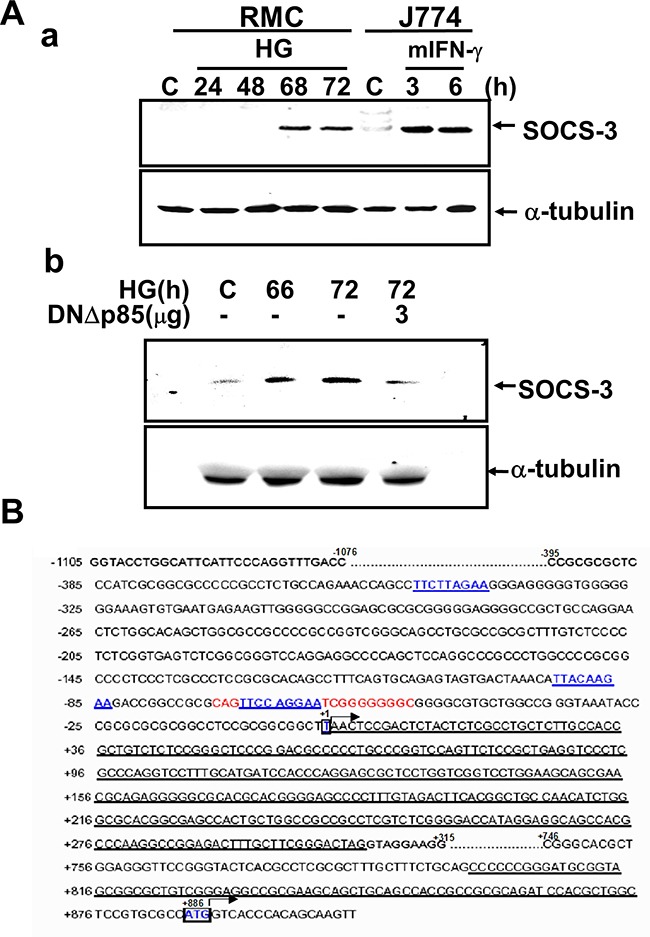
Induction of SOCS-3 expression in response to high glucose **A**. RMCs were stimulated with high glucose (HG) for the indicated times in the absence (a) or presence (b) of transfected dominant negative p85 vector (DNΔp85). J774 macrophage cells were treated with 100 units/ml IFN-γ as a positive control for SOCS-3 induction (a). Data representative are presented at least five independent experiments. **B**. Nucleotide sequence of the full-length ≈ 2.8-kb genomic 5′ region of rat SOCS-3. Exons are single underlined. The transcription start site is defined as +1. The translation initiation codon ATG and a putative TATA-box are indicated in bold letters. Two putative STAT binding elements are indicated in bold and underlined. The complete available sequence of the genomic 5′ region of rat SOCS-3 has been deposited in GenBank (accession no. AFJ249240).

We next assessed the activation of SOCS-3 through the PI3K pathway during HG-induced ECM accumulation. HG markedly increased the protein expression of SOCS-3 in RMCs at 66-72 h after HG treatment (Figure 3A). DNΔp85 transfection could abolish HG-induced SOCS-3 protein expression (Figure 3A–3b).

**Figure 4:**
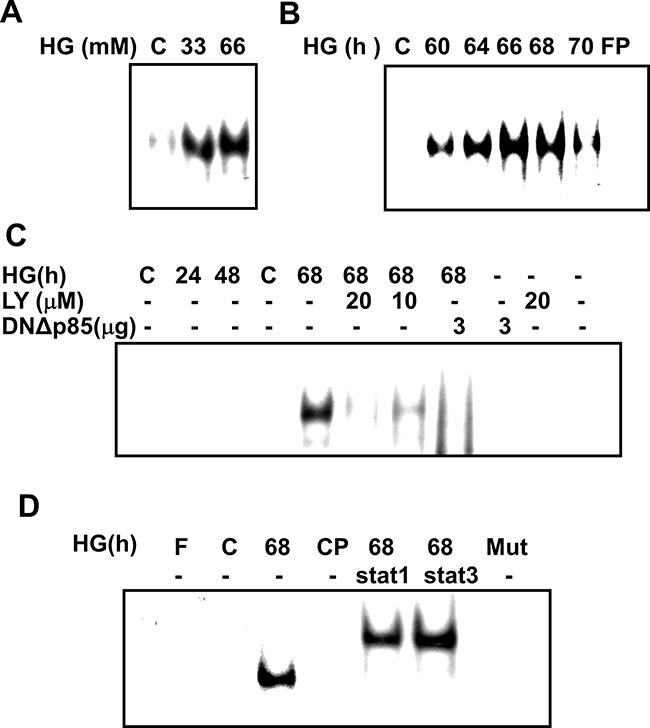
Time-dependent response of high glucose-induced SOCS-3 binding activation in RMCs Ten microgram of nuclear extracts from RMCs, either untreated or stimulated with high glucose (HG), were incubated with ^32^P-labeled SOCS-3probes. Nuclear proteins were prepared and subjected to electrophoretic mobility shift assay (EMSA) as described in Materials and Methods. **A**. A representative EMSA under different concentrations ofHG (33 and 66 mM) for 68 h. **B**. Nuclear lysates of RMCs were stimulated for the indicated times with 33 mM HG. **C**. HG-induced SOCS-3 binding complexes in RMCs in the presence or absence of LY294002 or dominant negative p85 vector (DNΔp85). **D**. RMCs were treated with HG, and then nuclear extracts were pre-incubated with specific antibodies against STAT1 and STAT3, and subsequently mixed with ^32^P-labeled SOCS-3 or SOCS-3 mutant (Mut) probes. DNA-protein complexes were analyzed in nondenaturing acrylamide gels and revealed by autoradiography. In competition experiments (CP), 100-fold excess unlabeled competitor oligonucleotides were added to the preincubation reaction with the ds oligonucleotide. Data representative are presented at least five independent experiments.

To identify the region(s) responsive to HG in the SOCS-3 gene, we carried out a functional analysis of the SOCS-3 promoter binding activity. Nucleotide sequence of the full-length about 2.8-kb genomic 5′ region of rat SOCS-3 was shown in Figure 3B. Electrophoretic mobility shift assay (EMSA) showed a specific binding of nuclear extracts from HG-treated RMCs to a ds oligonucleotide probe spanning nucleotides –72 to –46 (STAT oligo) including the STAT1/STAT3 binding element from –69 to –61 (Figure 3B). Accordingly, the results of EMSA revealed that a strong DNA-binding activity to the oligonucleotide probe containing the SOCS-3 sequence was activated in RMCs under HG treatment (Figure 4). A time-response study indicated that HG effectively activated DNA binding activity early at 60 h and gradually increased at 64-68 h and then decreased at 70 h (Figure 4B). Moreover, pretreatment of cells with LY294002 or DNΔp85 transfection entirely abolished the HG-increased SOCS-3 promoter binding activity (Figure 4C). Mutation of the SOCS-3 consensus sequence resulted in the complete loss of DNA-binding activity (Figure 4D), suggesting that SOCS-3 induction by HG relies on a direct and specific binding of factor(s) to the SOCS-3 sequence. Addition of cold consensus oligonucleotide (100-fold in excess) completely abolished the mobility shift band, demonstrating the specificity of the protein/DNA interaction. Moreover, pre-incubation of nuclear extracts from HG-treated renal mesangial cells with antibodies against STAT1 and STAT3, fully displaced the DNA:protein complex (Figure 4D), indicating that the translocation factor(s) binding to the SOCS-3 motif is represented by STAT uniquely.

### HG induces the STAT1/3 phosphorylation and p27^Kip1^ up-regulation

**Figure 5:**
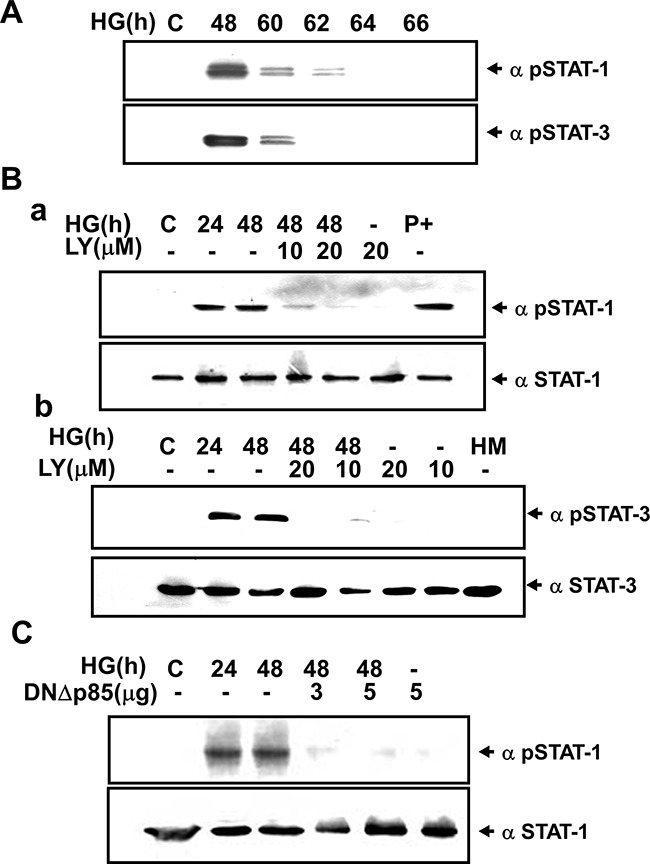
High glucose-induced phosphorylation ofSTAT1/3 in renal mesangial cells requires PI3K **A**. Induction of STAT1/STAT3 phosphorylation in response to high glucose (HG). RMCs were stimulated with HG for the indicated times. **B**. RMCs were incubated with HG in the presence or absence of LY294002 (LY, 10-20 μM) for 24 and 48 h. “P+” is the positive control for Hep 3B cells treated with IFN-γ 500 U/ml for 30 min. **C**. Cells were transiently transfected with dominant negative p85 vector (DNΔp85). Quiescent RMCs were incubated with or without HG for 24 and 48 h. Results shown are representative of three independent experiments.

**Figure 6:**
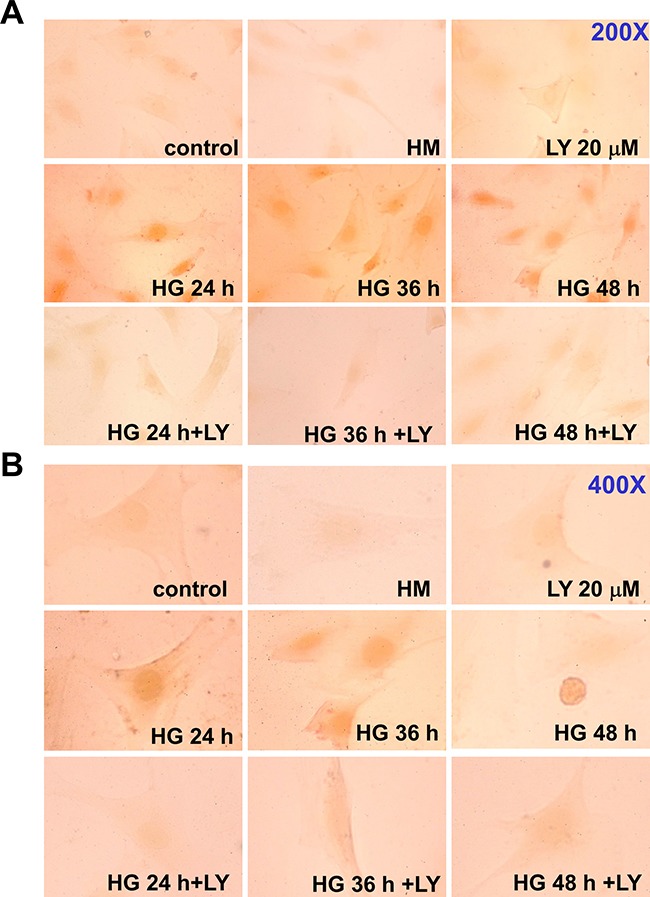
Effect of high glucose on STAT1 phosphorylation in RMCs The STAT1 phosphorylation and nuclear translocation in high glucose (HG)-treated RMCs were analyzed by immunocytochemistry as described under “Materials and Methods”. Cells were exposed to HG with or without LY294002 (LY, 20 μM) pretreatment for 30 min. Most of the nuclei in RMCs were positively stained at 24, 36, and 48 h after the treatment of HG. **A**. 200X magnify; **B**. 400X magnify.

SOCS-3 induction has been shown to be strictly dependent on both STAT1 and STAT3 activation in IFN-γ-stimulated J774 macrophage cells [19] (Figure 3A–3a), and on the STAT3 activation in G-CSF-activated granulocytes [20]. We next investigated the phosphorylation of STAT1 and STAT3 in HG-treated RMCs determined by Western blotting and immunocytochemistry. Western blotting analysis revealed that HG induced the tyrosine phosphorylation of STAT1 (Tyr701) and STAT3 (Tyr705) in a time-dependent manner (Figure 5A). LY294002 and dominant negative DNp85 vector transfection effectively prevented the effect of HG on the STAT1 and STAT3 phosphorylation (Figure 5B and 5C). In fact, the STAT phosphorylation was declined at 60-66 h after the treatment of HG (Figure 5A), which was consistent with the initial time for the induction of SOCS-3 expression. Moreover, immunocytochemical analysis also showed that treatment with HG for 24-48 h markedly increased the staining for phosphorylated STAT1 in RMCs, which could be inhibited by LY294002 (Figure 6).

**Figure 7:**
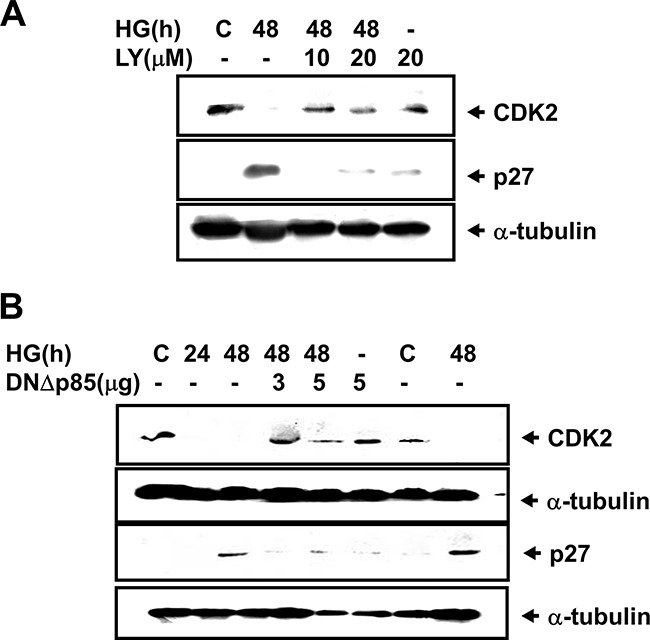
High glucose down-regulated CDK2 expression and up-regulated p27^Kip1^ expression in RMCs **A**. Renal mesangial cells were incubated with or without 10 or 20 μM LY294002 for 48 h in medium containing either normal glucose (5.5 mM) or high glucose (HG, 33 mM). **B**. Cells were transiently transfected with dominant negative p85 vector (DNΔp85). Quiescent renal mesangial cells were incubated with or without high glucose for 24 and 48 h. Results shown are representative of four independent experiments.

The expression of glomerular fibronectin, an ECM protein, has been shown to be increased in diabetic wild-type mice, but did not increase in diabetic p27^Kip1^-knockout mice [21]. We next investigated the role of PI3K and STAT1/3 in the cyclin kinase inhibitor p27^Kip1^ protein expression in HG-treated mesangial cells. Exposure of RMCs with HG for 48 h markedly decreased the CDK2 protein expression and increased the p27^Kip1^ protein expression, which could be dramatically inhibited by both LY294002 treatment and DNΔp85 transfection (Figure 7A and 7B). Transfection of both DNΔSTAT1 and DNΔSTAT3 could also inhibit the increased p27^Kip1^ protein expression in HG-treated mesangial cells (Supplementary Figure 1).

## DISCUSSION

**Figure 8:**
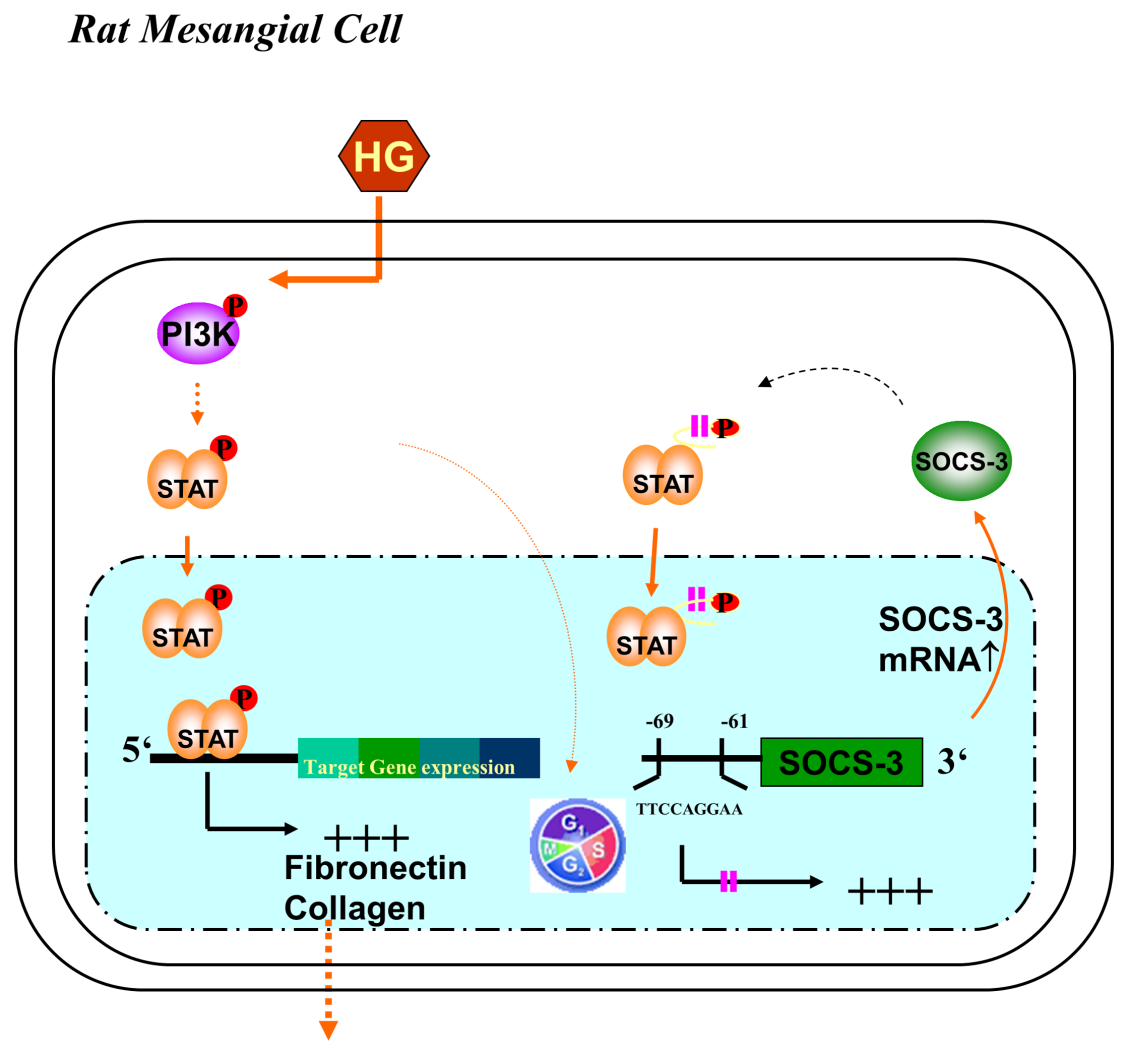
Schematic representation of proposed intracellular signaling leading to high glucose-induced extracellular matrix formation in RMCs The high glucose (HG)-induced signaling cascade in the RMCs involved with tyrosine phosphorylation of PI3K, STAT1/3, and SOCS-3. The SOCS-3 promoter has a functionally critical STAT1/3 binding region at –69 to –61, individually. By PI3K/STAT signaling, SOCS-3 probably was regulated by HG-induced mesangial cell gene expression and also exerts a negative feedback on its interrelated gene expression.

The responses to glomerular and tubulointerstitial cell injury in most forms of renal diseases includes changes in cell number (proliferation and apoptosis) and cell size (hypertrophy); these events typically precede and may be responsible for the accumulation of ECM proteins that leads to a decrease in renal function [22]. Mesangial cell induced excessive depositions of ECM in the kidney are indeed manifestations of diabetic nephropathy. Previous studies have demonstrated that PI3K is an important signaling mediator of mesangial cell proliferation and hypertrophy *in vitro* [7, 8]. High glucose has also been shown to transactivate PI3K, leading to cell proliferation and ECM accumulation in mouse mesangial cell line MES-13 [23]. However, the role of PI3K in the diabetic glomerular ECM formation still needs to be clarified. In the present study, we showed a number of findings with respect to the molecular mechanism of hyperglycemia-induced and PI3K-dependent regulation of the STAT1/3 and SOCS-3 signals, which involved in the cascade of sequential reactions to induce a large amplification of the signal in ECM formation in primary RMCs. Schematic representation of proposed intracellular signaling leading to HG-induced ECM formation in RMCs was shown in Figure 8. First, pathological levels of glucose activated PI3K, resulting in a modulation of STAT1/3 phosphorylation. Second, the signaling cascades, which contributed to overproduction of fibronectin and collagen, were essential for the inhibition of the HG-induced STAT1/3 activation by PI3K inhibitor and DNp85. And finally, HG strictly induced SOCS-3 protein expression in a PI3K-dependent manner. HG-increased SOCS-3 expression was associated with STAT1/3, suggesting a possible innovative transcriptional mechanism for hyperglycemia-induced ECM accumulation. In addition to our findings, Ray S et al. also demonstrated the endogenous STAT3 target gene, SOCS3, was upregulated in hypertrophic scar fibroblasts (HSFs) and showed increased STAT3 binding on its promoter. A STAT3 peptide inhibitor abrogated fibronectin and α2(I) collagen gene expression in HSFs indicating involvement of STAT3 in ECM production [24]. Furthermore, increasing evidence suggests that hyperglycemia may promote tumor progression [25]. Blood glucose level in colorectal cancer patients correlates significantly with local tumor malignancy [26]. Lin CY et al. demonstrated that high glucose-induced migration and invasion of CT-26 rat colorectal cancer cells were obviously contributed by STAT3-activated ECM remodeling enzyme MMP9 expression [27]. As dynamic remodeling of ECM plays an important role in regulating cell behavior as well as tumorigenesis, blood sugar regulation is a critical issue of concern and deserves further investigation. Unlike high glucose, the addition of high mannitol to the media did not display the responses in RMCs, indicating that the high glucose-triggered responses are not the result of high osmolality within the media. HG caused the increase in mRNA and protein expressions of mesangial matrix, including fibronectin [4, 28] and α1(IV) collagen [29]. These responses for ECM formation in mesangial cells could be triggered by angiotensin II [30], endothelin-1 [29, 31], and PDGF [32]. Inhibition of the Jak/STAT signaling pathway has been demonstrated to prevent the HG-induced increase in TGF-β and fibronectin synthesis in mesangial cells [4]. In the present study, results showed that DNΔp85 transfection or LY294002 entirely abrogated HG-enhanced STAT1 and STAT3 phosphorylation and fibronectin and α1(IV) collagen protein expression in RMCs, indicating that PI3K activation is required for HG-enhanced STAT1/3 activation and ECM protein expressions.

The SOCS family of cytokine-inducible proteins constitutes a negative feedback loop of JAK/STAT pathway [9, 10]. Accumulated evidence has shown that SOCS proteins can potently block the Jak/STAT signaling pathway in the pathogenesis of various inflammatory diseases [16, 33, 34]. It has been shown that the expressions of SOCS family genes, mainly SOCS-3, are markedly increased in the kidneys of experimental models of immune complex glomerulonephritis [16]. SOCS proteins are known to possess the ability to negatively regulate the activation of STAT proteins [35, 36]. Delivery of SOCS1 and SOCS3 genes to the diabetic kidneys has been demonstrated to inhibit the renal STAT1 and STAT3 activities and proinflammatory and profibrotic protein expressions [18]. In the present study, we further found that both LY294002 treatment and DNΔp85 transfection effectively inhibited the HG-triggered SOCS-3 DNA binding activity in primary RMCs. The STAT1/3 phosphorylation was increased at 24-48 h after the treatment of HG. The HG-activated STAT1/3 activation was declined at 60-66 h, which was consistent with the initial time for the induction of SOCS-3 expression, indicating a negative regulatory role of SOCS-3 in STAT1/3 activation in HG-treated mesangial cells. SOCS-3 induction by HG was achieved via phosphorylation of STAT1/3, which in turn binds to the SOCS-3 element through a PI3K-activated signaling pathway. Because we focused on the HG-induced SOCS-3-regulated STAT1/3-related signaling, the direct effect of SOCS-3 knockdown on the ECM production was not demonstrated in the current work. However, we highly suggest that PI3K is involved in the SOCS-3-regulated STAT1/3-related signaling pathway to regulate ECM formation in RMCs under HG exposure, and further studies are warranted.

A previous study has shown that both hyperplasia and hypertrophy participate to approximately same extent in causing kidney enlargement in streptozotocin-induced diabetic rats [37]. Cell cycle has been demonstrated to be regulated in diabetic nephropathy [38]. Cyclin kinase inhibitor p27^Kip1^, which was up-regulated in glomeruli of diabetic animals and HG-treated mesangial cells, has been suggested to be required for hyperglycemia-induced glomerular or mesangial cell hypertrophy [3, 39]. Awazu et al. have shown that mesangial expansion was milder and glomerular hypertrophy did not develop and glomerular ECM protein expression of fibronectin did not increase in diabetic p27^Kip1^ knockout mice [21]. In the present study, we found that HG markedly decreased CDK2 protein expression and increased p27^Kip1^ protein expression in RMCs, which could be inhibited by PI3K inhibitor or dominant negative p85 vector transfection. These results indicate that a PI3K signaling is involved in the HG-regulated p27^Kip1^ expression in RMCs, and imply that the induction of PI3K-regulated p27^Kip1^ pathway may contribute to the accumulation of ECM during hyperglycemia; nevertheless, the further studies are needed to clarify this possibility of its therapeutic potential in the future.

In conclusion, this study demonstrates that PI3K up-regulates SOCS-3 expression/DNA binding activity, STAT1/3 activity, and ECM formation in primary RMCs under HG exposure. SOCS-3 expression induced by HG is achieved via STAT1/3 activation, and the induced SOCS-3 signaling subsequently down-regulates STAT1/3 activation in mesangial cells.

## MATERIALS AND METHODS

### Cell isolation and culture

Renal mesangial cells were isolated from collagenase-treated rat glomeruli as previously described [4]. In brief, glomeruli were harvested from male Sprague-Dawley rats (150 to 200 g) by filtration with ice-cold 0.9% NaCl solution through a 200-, 150-, 120-, and 50-μm nylon mesh. Those retained on the sieve were collected and washed by centrifugation (4°C, 2000 *g*), and incubated with 250 units/ml collagenase (type I) for 30 min at 37°C under constant and gentle shaking. Mesangial cells were plated on plastic tissue culture flasks in low glucose DMEM (pH 7.4) either with normal glucose (NG, 5.5 mM) or with HG (33 mM) or mannitol (33 mM) concentrations. The culture medium was supplemented with 10% fetal bovine serum, 100 U/ml penicillin, 100 μg/ml streptomycin, and 25 μg/ml amphotericin (an antimycotic agent). Cells were routinely passaged by trypsinization after they reached 80 % confluency using 10 cm culture dishes and incubating them at 37°C in a humidified chamber with a 5% CO_2_-95% air mixture. Cells were subcultured at 1:6 at 7-day intervals, and the medium was changed at 2-day intervals. Cells at passages 1–6 were grown to 75–85% confluence, washed once with serum-free DMEM, and growth-arrested in serum-free DMEM in NG for 24 h to synchronize the cell growth. In some experiments for detection over 48 h, starvation medium was added 2% fetal bovine serum (FBS) to maintenance for optimal cell condition.

### Phosphoinositide 3-kinase activity assay

PI3K activity was determined as described previously [7]. Briefly, RMCs cultured in 6-well dishes were serum-deprived for 24 h, 2 x10^5^ cells received different treatments and were washed twice with ice cold phosphate-buffered saline and lysed with 1 mM lysis buffer (137 mM NaCl, 2.7 mM KCl, 1 mM MgCl_2_, 1 mM CaCl_2_, 1% Nonidet P-40, 10% glycerol, 1mg/ml bovine serum albumin, 20 mM Tris, pH 8.0, 2 mM orthovanadate). Cell extracts were incubated with 2 μg of anti-p85 antibody overnight at 4°C. The immunocomplex was precipitated with 50 μl of protein A-Sepharose for 1 h at 4°C and washed three times with lysis buffer, twice with LiCl buffer (0.5 M LiCl, 100 mM Tris, pH 7.6), and twice with TNE buffer (10 mM Tris, pH 7.6, 100 mM NaCl, 1 mM EDTA). The immunocomplex was preincubated with 10 μl of 20 mM Hepes (pH 7.4), containing 2 mg/ml propidium iodide (Sigma) on ice for 10 min. Kinase reaction was performed by adding 40 μl of reaction buffer (10 μCi of [γ-^32^P]ATP, 20 mM Hepes, pH 7.4, 20 μM ATP, 5 mM MgCl_2_) at room temperature for 15 min. Added 100 μl of 1 M HCl extracted with 200 μl of a 1:1 mixture of chloroform and methanol to stop the reaction. The radiolabeled lipids were separated by thin-layer chromatography and visualized by phosphorimaging.

### Immunoblotting

The cellular lysates were prepared and a 100 μg sample of each lysate was subjected to electrophoresis on 10-15% SDS-polyacrylamide gels. The samples were then electroblotted on polyvinylidene difluoride membranes. After blocking, blots were incubated with anti-fibronectin (Transduction Laboratories), anti-collagen α IV (Santa Cruze Biotechnology), anti-STAT1, anti-STAT3, anti-phospho-STAT1, and anti-phospho-STAT3 (New England BioLabs) antibodies in PBST (phosphate-buffered saline within 0.1% Tween 20) for 1 h followed by two washes (15 min each) in PBST. The membranes were then incubated with horseradish peroxidase-conjugated secondary antibodies for 30 min. Enhanced chemiluminescence reagents (Amersham) were employed to depict the protein bands on membranes.

### Cell transfection

RMCs (5 × 10^5^) were seeded in complete medium, and followed by serum starvation 16 h before transfection. Transfection was performed using the lipofectin reagent (Invitrogen) according to the recommendations of the manufacturer. Cells were transfected with 2 μg of plasmids containing the DN-p85 kindly provided by Dr. Lin WW (Institute of Pharmacology, National Taiwan University, Taipei, Taiwan) or pcDNA3 control vector. The efficiency of transfection (about 80%) was determined using an equal amount of a plasmid encoding the green fluorescent protein under the cytomegalovirus promoter. The percentage of fluorescent cells was determined 48 h after transfection by flow cytometry and fluoromicroscope.

### Preparation of nuclear extracts

Nuclear extracts were prepared as described previously [7]. Approximately 2×10^6^ cells were harvested in cold PBS, pH 7.4, and pelleted at 1200 rpm for 5 min. The pellets were resuspended in 400 μl of cold buffer A (10 mM HEPES, pH 7.9 at 4°C, 1.5 mM MgCl_2_, 10 mM KCl, 0.5 mM DTT, 0.2 mM PMSF), and incubated for 10 min on ice. The samples were centrifuged for at 1000 rpm for 2 min. The supernatants were discarded and the pellets were resuspended in 100 μl of cold buffer C (20 mM HEPES, pH 7.9, 25% glycerol, 420 mM NaCl, 1.5 mM MgCl_2_, 0.2 mM EDTA, 0.5 mM DTT, 0.2 mM PMSF) and incubated on ice for 30 min. After centrifugation at 1400 rpm for 10 min, the supernatants were frozen at -80°C. Protein concentration was determined using a colorimetric assay by a BCA Protein Assay Kit (PIERCE).

### Electrophoretic mobility shift and supershift assay

EMSA were performed as described previously [40]. The nuclear extracts (5 μg) were incubated with a ^32^P-labeled double-stranded oligonucleotide probe containing the STAT-binding element (SOCS-3) located at -69/-61 nucleotides of the promoter of the SOCS-3 gene. The following oligonucleotide with the SOCS-3 consensus binding sequence was used: 5-CAGTTCCAGGAATCGGGGGGGC-3′ or with the mutant form of this sequence 5′-CAGTTCCAGGTTTCGGGGGGGC-3′ for 15 min. The consensus oligonucleotide probes were end-labeled using T4 polynucleotide kinase and [γ-^32^P] ATP in a binding reaction buffer. For binding reaction, 2 ng of the labeled oligonucleotide (approximately 20,000 cpm) and 2 μg of poly dIdC (Amersham) carrier were incubated with 20 μg of nuclear protein in a binding buffer (10 mM HEPES, 60 mM KCl, 1 mM DTT, 1 mM EDTA, 7% glycerol, pH 7.6) for 30 min at room temperature. Samples were loaded on 6 % polyacrylamide nondenaturing gel and electrophoresed for 3 h at 180 V in 0.5 × TBE buffer and visualized by autoradiography. For competition experiments, a 100-fold excess of the unlabeled oligonucleotides was added 15 min before incubation of nuclear extracts with the end-labeled oligonucleotides. Moreover, the supershift experiments were performed by incubating extracts with 2 μg of anti-STAT1or anti-STAT3 antibodies (New England BioLabs) for 60 min at room temperature before adding the labeled probe. The reaction mixtures were then subjected to electrophoresis in a 6% nondenaturing polyacrylamide gel, dried, and analyzed in autoradiography.

### Immunocytochemical staining

To detect the phosphorylation of STAT1 *in situ* and nuclear translocation, 80% confluent RMCs were seeded on the cover glass coated with 1 N for 15 min at 4°C. The cells were exposed to tested materials for 60 min, and then fixed in 4% paraformaldehyde. The samples were reacted with anti-phospho-STAT1 antibody (1: 1000 dilution in PBS, New England BioLabs) for over nigh at 4°C temperature. After three washes with PBS, bound primary antibody was detected with a biotinylated anti-rabbit antibody followed by peroxidase system. Peroxidase reaction was performed by using 3,3′-diaminobenzidine (Sigma) as a chromogenic substrate.

### Statistical analysis

The values given in this study are presented as means±SEM. All analyses were performed by ANOVA followed by a Fisher's least significant difference test. A P value of less than 0.05 was viewed as statistically significant.

## SUPPLEMENTARY FIGURE


