# Supplementary material for: Activation of PI3K in response to high glucose leads to regulation of SOCS-3 and STAT1/3 signals and induction of glomerular mesangial extracellular matrix formation
Source: Oncotarget. 2017 Jan 24;8(10):16925–38. doi: 10.18632/oncotarget.14808 (PMC5370011; doi:10.18632/oncotarget.14808)
Supplement: Supplementary file 1 [file oncotarget-08-16925-s001.pdf]

Activation of PI3K in response to high glucose leads to regulation of SOCS-3 and STAT1/3 signals and induction of glomerular mesangial extracellular matrix formation

SUPPLEMENTARY FIGURE

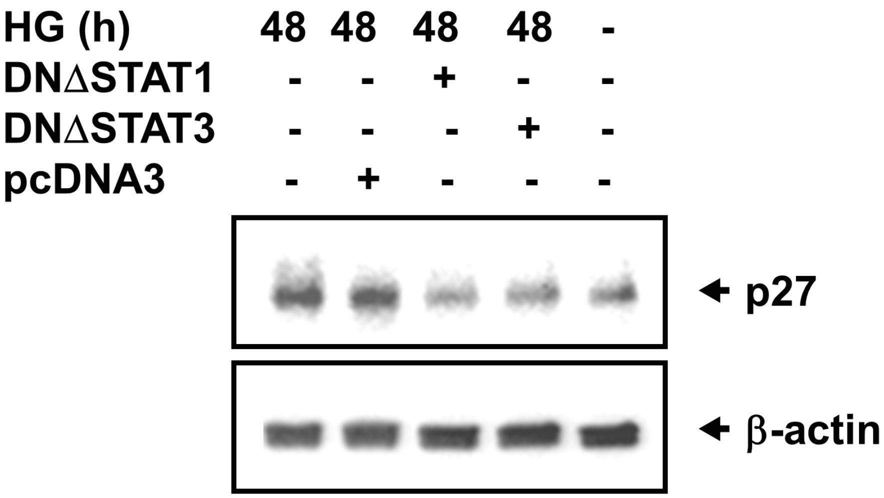

Supplementary Figure 1: STAT1/3 knockdown suppresses p27^{Kip1} protein expression in HG-treated mesangial cells. Exposure of RMCs with HG for 48 h markedly increased the p27^{Kip1} protein expression, which could be dramatically inhibited by transfection of both DNΔSTAT1 and DNΔSTAT3 in HG-treated mesangial cells.
